# Molecular Tracking of the *Leishmania* Parasite

**DOI:** 10.3389/fcimb.2021.623437

**Published:** 2021-02-22

**Authors:** Srija Moulik, Shilpa Sengupta, Mitali Chatterjee

**Affiliations:** Department of Pharmacology, Institute of Postgraduate Medical Education and Research, Kolkata, India

**Keywords:** anti-leishmanial antibodies, biomarkers, HIV-VL, kinetoplast DNA kDNA, molecular diagnosis, Post Kala-azar Dermal Leishmaniasis PKDL, Visceral Leishmaniasis VL

## Abstract

With the Visceral Leishmaniasis/Kala-azar Elimination Program in South Asia in its consolidation phase, the focus is mainly on case detection, vector control, and identifying potential sources of infection. Accordingly, emphasis is presently on curbing transmission, which is potentially achievable by identification and elimination of potential reservoirs. The strongest contenders for being the disease reservoir are cases of Post Kala-azar Dermal Leishmaniasis (PKDL) which occurs in a minor proportion of individuals apparently cured of Visceral Leishmaniasis (VL). The demonstration of parasites in tissue aspirates despite being a risky and invasive process is the gold standard for diagnosis of VL, but is now being replaced by serological tests e.g., rK39 strip test and direct agglutination test. However, these antibody based tests are limited in their ability to diagnose relapses, detect cases of PKDL, and monitor effectiveness of treatment. Accordingly, detection of antigen or nucleic acids by polymerase chain reaction has been successfully applied for monitoring of parasite kinetics. This review article provides updated information on recent developments regarding the available antibody or antigen/nucleic acid based biomarkers for longitudinal monitoring of patients with VL or PKDL and emphasizes the need for availability of studies pertaining to quantification of treatment response or relapse.

## Introduction

Worldwide, an estimated 50,000 to 90,000 new cases of kala-azar or Visceral Leishmaniasis (VL), caused by the parasite *Leishmania donovani* occur annually, with the contribution of Bangladesh, India, and Nepal being around 67% (https://www.who.int/news-room/fact-sheets/detail/Leishmaniasis last accessed on 4th December, 2020). In a joint VL elimination initiative launched by the Governments of India, Bangladesh, and Nepal in 2005, the target for elimination by 2020 was aimed at reduction of the annual incidence of VL to below 1/10,000 persons at an upazilla level in Bangladesh, sub-districts [(block public health centre (PHC)] level in India and district level in Nepal (Sundar et al., 2018). This elimination was considered feasible owing to the defined and limited geographical spread of VL, absence of an animal reservoir, a single vector *Phlebotomus argentipes*, availability of an effective diagnostic test, use of an oral drug miltefosine which was later replaced by a single dose of liposomal amphotericin B (AmBisome), along with a strong political commitment (Hirve et al., 2017; Alves et al., 2018; Gedda et al., 2020).

The Kala Azar Elimination Programme (KAEP) consists of four consecutive phases and began with a “preparatory phase” which involved development/review of national policy, strategic and advocacy plans, operational plans to implement the national plan for elimination, development, and adoption of technical guidelines (Sundar et al., 2018). This was followed by a multiprolonged “attack phase” that included integrated vector management with indoor residual spraying for 5 years in affected areas along with active surveillance, early diagnosis and complete treatment. Till date, the number of VL cases in India, Nepal, and Bangladesh have declined steadily from over 77,000 reported cases in 1992 to fewer than 7,000 cases in 2016 (Rijal et al., 2019) and further reduced to 3,128 in 2019 (World Health Organization, 2020).

The KAEP has since moved into the consolidation phase, where ongoing active surveillance is aimed at detecting and treating potential disease reservoirs, namely asymptomatic cases of VL and patients with Post-kala-azar dermal Leishmaniasis (PKDL), the latter being a dermal aftermath in individuals apparently cured of VL. This consolidation phase will end when three years of active surveillance demonstrates no increase in the incidence rate at district/subdistrict/upazila levels in the endemic countries. Finally, a maintenance phase will be undertaken to ensure the case incidence is sustained at less than 1 per 10,000 population (World Health Organization, 2020).

As PKDL cases harbor parasites in dermal lesions that are easily accessible to the sandfly, it makes them key players in the transmission cycle, and bears the burden of being a major factor potentially capable of jeopardising the success of the KAEP (Duthie et al., 2019). However, tracking patients with PKDL is a formidable challenge owing to its low morbidity and practically no mortality. This accounts for their poor health seeking behavior, and ultimately translates into PKDL being a potent, mobile reservoir (Zijlstra et al., 2017; Burza et al., 2018). Furthermore, identifying biomarkers in PKDL is hampered by the lack of adequate studies along with a considerable heterogeneity, reiterating the need for well-designed trials to assess diagnostic accuracy (Adams et al., 2013). In addition, as the pathophysiology of PKDL is different from VL or other cutaneous leishmaniases and even differs based on geographical locations, one cannot extrapolate from existing biomarkers of *Leishmania* infection (Kip et al., 2015; Zijlstra et al., 2017).

With the availability of effective diagnostic tools and treatments for VL and PKDL cases as also integrated vector management, countries have progressed towards the elimination goal. A lingering concern is that this lowering of case numbers can lead to a decreasing awareness within the communities and health care providers, and result in cases of VL and PKDL being ignored or missed, resulting in resurgence. It is important that such a scenario be averted, emphasizing the need for objective quantification of the infection burden. The availability of molecular based tools could facilitate development of rapid and high throughput approaches to detect parasites. Accordingly, this review focuses on the recent developments regarding antibody or antigen/nucleic acid based biomarkers with potential for longitudinal monitoring of patients with VL or PKDL, and highlights the limited availability of studies pertaining to quantification of treatment response and/or relapse.

### Ethics

Written informed consent was obtained from the individual(s) and/or minor(s)’ legal guardian/next of kin for the publication of any potentially identifiable images or data included in this article.

### Strategy for Literature Search

**Figure 1:**
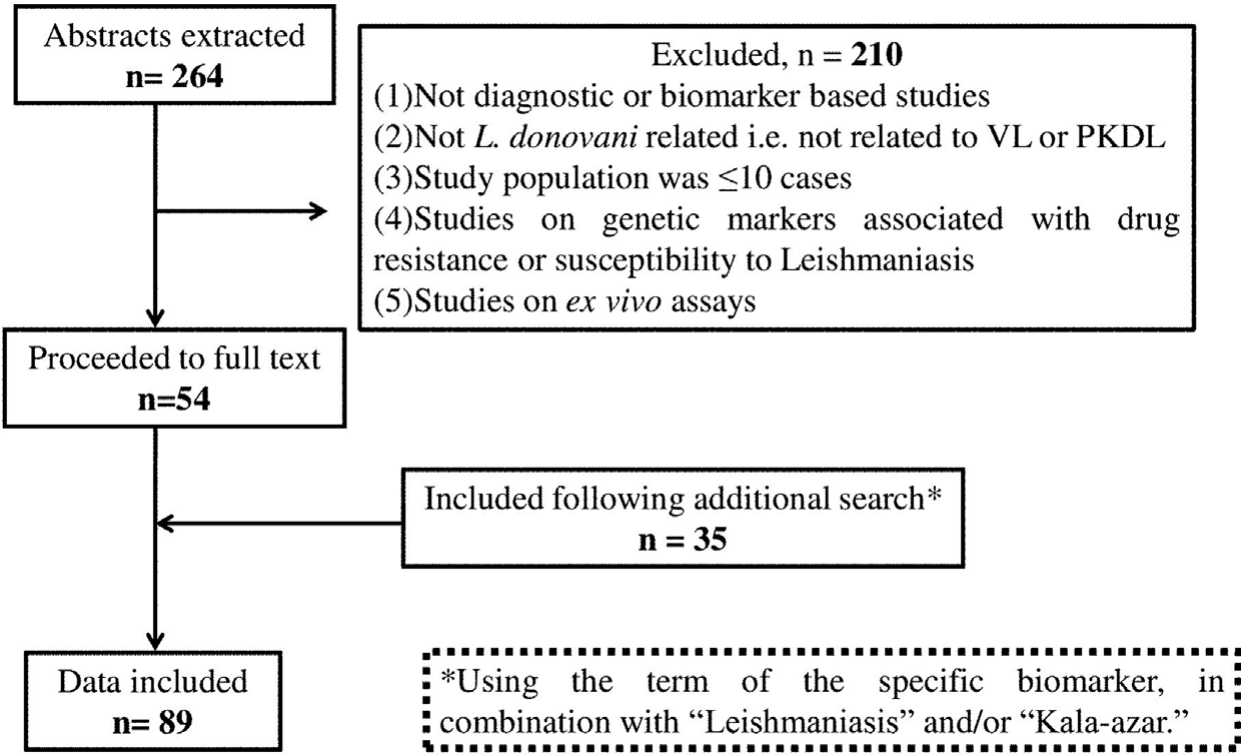
Flow diagram of the studies included.

The potential biomarkers for VL and PKDL were identified *via* the PubMed database pertaining to publications after 2000 and restricted to the English language, using the following key words: “(((Visceral Leishmaniasis-[title]) or Kala -azar[title]) or PKDL[title]) and (((((((((biomarker) or biomarkers) or marker) or diagnosis) or markers) or level) or levels) or concentration) or activity) or profile) or *Leishmania* antigen/antibody based test)”. The date last searched was 30^th^ September, 2020 and publications that did not focus on the identification or evaluation of biomarkers as a diagnostic approach were excluded (**Figure 1**). Additionally, secondary literature was included based on references included in the identified primary literature.

### Evaluation Criteria

The translational potential of serological and nucleic acid biomarkers was based on five criteria: (i) Assay sensitivity, the marker’s quantification in patients at disease presentation (ii) specificity, in relation to co-endemic infectious diseases, such as malaria, tuberculosis, enteric fever and HIV for VL, and in case of PKDL, diseases like leprosy, psoriasis and vitiligo, (iii) advantages, (iv) limitations/disadvantages, and (v) ability to monitor responses to anti-leishmanial therapy

## Results and Discussion

### Literature Search

The primary literature search identified 264 studies for which the titles were screened and assessed for relevance based on key words “(((Leishmaniasis-[title]) or Kala -azar[title]) or PKDL[title]) and (((((((((biomarker) or biomarkers) or marker) or diagnosis) or markers) or level) or levels) or concentration) or activity) or profile) or PCR) or qPCR)”, **Figure 1**. Abstracts (n = 210) were considered as non-relevant (**Figure 1**) and thereafter, full texts of the remaining studies were assessed for their relevance. This translated into 54 publications being included in this review. Additionally, 35 more studies were identified following a secondary literature search, i.e., using the search keywords as the specific biomarker(s) in combination with “Leishmaniasis” and/or “Kala-azar”, accounting for a total of 89 studies. The biomarkers studied in these patients with VL and PKDL were sub-grouped based on (i) circulatory markers or (ii) nucleic acid markers. Accordingly, for VL and PKDL, nine and six circulatory markers respectively were identified (**Tables 1**, **4**). In case of nucleic acid based markers, eight and five were identified for VL and PKDL, respectively (**Tables 2**, **5**).

**Table 1:** Antigen/Antibody Markers of VL.

Methods	Target	Biological source	Pre T/tAbsorbances/titres	Post T/tAbsorbances/titres	Sensitivity (%)	Specificity (%)	Reference
Latex agglutination (KAtex)	Carbohydrate antigen	Urine, n = 382 (Pre); n = 273 (Post)	332/382 (86.9%)	8/273: (3%)	87	99	Sundar et al., 2006
		Urine, n = 230	169/230 (73.5%)	ND	73.5	94	Sundar et al., 2007
		Urine, n = 36	27/36 (75%)	ND	75	100	Salam et al., 2011
		Urine, n = 49	28/49 (57.1%)	ND	41.7–73.4	84.3	Diro et al., 2007
		Urine, n = 35	18/35 (51.4%)	ND	51.4	98.3	Ben-Abid et al., 2017
		Oral fluid, n = 35	28/35 (80%)	ND	80	88.3	-do-
	A2 amastigote antigen (A2 LAT)	Blood, n = 43	38/43 (88%)	ND	88	93.5	Akhoundi et al., 2013
	Promastigote antigen (Pro LAT)	-do-	38/43 (88%)	ND	88	91	-do-
RBC ELISA	9-O-acetylated sialic acids	Blood, n = 56	54/56 (96.4%)	ND	96	79	Chava et al., 2002
PEG ELISA	*Leishmania* promastigote (IgG and IgG subclasses containing Immune complex)	Blood, n = 24 (as confirmed by rk39)	IgG: 23/24: 1.73 ± 0.11	ND	IgG = 96	IgG = 96.4	Datta et al., 2015
		-do-	IgG1:12/24: 0.32 ± 0.05	ND	IgG1 = 50	IgG1 = 100	-do-
		-do-	IgG2:7/24: 0.44 ± 0.06	ND	IgG2 = 29	IgG2 = 100	-do-
		-do-	IgG3:8/24: 0.69 ± 0.04	ND	IgG3 = .33.3	IgG3 = .100	-do-
Enzyme-linked Immunosorbent Assay (ELISA)	*Leishmania* promastigote (IgG subclasses)	Blood, n = 38 (Adults, n = 24 & Children, n = 14)	IgG: Adults, 1.58 ± 0.61 Children: 1.44 ± 0.61	ND	ND	ND	Ansari et al., 2008
		-do-	IgG1, Adults: 0.61 ± 0.31; Children: 0.61 ± 0.25	ND	ND	ND	-do-
		-do-	IgG3, Adults: 0.16 ± 0.11 Children 0.28 ± 0.14	ND	ND	ND	-do-
		-do-	IgG4, Adults: 0.15 ± 0.06 Children 0.25 ± 0.02	ND	ND	ND	-do-
		Blood, n = 66 (Pre); n = 49 (Post)	IgG1: 60/66 (90.9%)	IgG1:7/49 (14%)	90	ND	Bhattacharyya et al., 2014
		-do-	IgG3: 50/66 (75.7%)	IgG3: 7/49 (14%)	75	ND	-do-
		n = 54 (Relapsed)	NA	Relapsed VL			
				IgG1:45/54 (83.3%)	84.2	ND	-do-
				IgG3:29/54 (53.7%)	52.6	ND	-do-
		n = 80 as confirmed by rk39	IgG1: 72/80 (90%)	ND	IgG1: 90	IgG: 77.2	Freire et al., 2019
		-do-	IgG3:60/80 (75%)	ND	IgG3: 75	ND	-do-
		-do-	IgG: 66/80 (82.5%)	ND	IgG: 77	ND	-do-
	Gp63, EF1α, and cysteine protease C (CPC)	Blood, n = 54 (Pre and Post t/t)	Gp63:50/54 (92.5%)	No significant changes found	92.5	60–95.2	Didwania et al., 2020
		-do-	EF1 α: 52/54 (96.2%)		96.2	10–100	-do-
		-do-	CPC: 53/54 (98.1%)		98.1	65–100	-do-
		Urine, n = 50 (Pre and Post t/t)	Gp63:45/50 (90%)	Levels declined sharply at	90	88.8–100	-do-
		-do-	EF1 α: 42/50 (84%)	6 months post treatment	84	55.5–94.7	-do-
		-do-	CPC: 48/50 (96%)		96	94.4–100	-do-
	rK39	Blood, n = 285	284/285 (99.9%)	ND	96.3–99.6	75.0–88.3	Machado de Assis et al., 2012
		Blood, n = 84	72/84 (85.7%)	ND	85.7	86	Hosseini Farash et al., 2020
	BHUP1	Blood, n = 108	103/108 (95.3%)	ND	95	96–100	Kumar et al., 2012
	Li-rK39 (*Leishmania infantum* rK39)	Blood, n = 100	96/100 (96%)	ND	96.8	93.8%	Rezaei et al, 2019
	rEnolase	Blood, n = 30	30/30 (100%)	ND	83–100	92.3–99	Duarte et al., 2017
	rA2	-do-	28/30(94%)	ND	75.1–99.8	62.4–83.9	-do-
	SLA	-do-	15/30(50%)	ND	27.2–72.8	92.3–99.6	-do-
	rKDDR	Blood, n = 84	74/84 (88.5%)	ND	88.5	97.3	Dhom-Lemos et al., 2019
	rEF1b	Blood, n = 30 Cut off level rEF1b >0.4135	30/30 (100%)	ND	100	100	Santos et al., 2019
	rk28	Blood, n = 252	251/252 (99.6%)	ND	99.6	94.7–100	Vaish et al., 2012
	rLiHyS	Blood, n = 23	23/23 (100%)	ND	94.7–`100	93.8–100	Dias et al., 2018
	40S ribosomal protein S12 antigen	PBMC, n = 14 confirmed by rk39 and PCR	9/14 (64.3%)	ND	68	ND	Zhang et al., 2018
	LiHyC and PeptC	Blood, n = 30	LiHyC = 30/30 (100%)	ND	100	ND	Machado et al., 2020
		-do-	PeptC = 30/30 (100%)	ND	100	ND	-do-
	Li-isd1, Li-txn1, Li-ntf2, Ld-mao1, Ld-ppi1, and Ld-mad1	Urine, n = 24	Li-isd1 8/24	ND	33.3	ND	Abeijon et al., 2020
		-do-	Li-txn1 11/24	ND	45.8	ND	-do-
		-do-	Li-ntf2 7/24	ND	29.1	ND	-do-
		-do-	Ldmao 10/24	ND	41.6	ND	-do-
		-do-	Ld-ppi1 7/24	ND	29.1	ND	-do-
		-do-	Ld-mad1 0/24	ND	0	ND	-do-
	Ld-mao1 and Ld-ppi1	Urine, n = 45	Ld-mao1 20/45	ND	44.2	ND	Abeijon et al., 2019
		-do-	Ld-ppi1 13/45	ND	28.8	ND	-do-
	C1, C8, and C9	Blood, n = 80	C1 = 53/80 (66.2%)	ND	66.2	60	Fonseca et al., 2014
		-do-	C8 = 47/80 (58.2%)	ND	58.2	50	-do-
		-do-	C9 = 54/80 (67.9%)	ND	67.9	77.7	-do-
	rHRF, rLiHyD, rLiHyT, rLiHyV, rLiHyp6, rA2, or SLA	Blood, n = 30	rHRF = 30/30 (100%)	ND	100	96.7	Portela et al., 2018
		-do-	rLiHyD = 30/30 (100%)	ND	100	96.7	-do-
		-do-	rLiHyt = 30/30 (100%)	ND	100	96.7	-do-
		-do-	rLiHyV = 21/30 (70%)	ND	70	96.7	-do-
		-do-	rLiHyp6 = 10/30(33.3%)	ND	33.3	96.7	-do-
		-do-	rA2 = 16/30 (53%)	ND	53	96.7	-do-
		-do-	SLA = 14/30(46.7%)	ND	46.7	96.7	-do-
	Neopterin	Blood, n = 96 (Pre & Post t/t)	96/96 (100%)	4/96 (4%)	100	75	Kip et al., 2018
	Adenosine Deaminase (ADA)	Blood, Pre, and Post t/t; n = 39	Significantly higher than healthy controls	Significantly reduced by 3-fold	ND	ND	Vijayamahantesh et al., 2016
Immuno chromatographic test (ICT)	rK39	Blood, n = 150	149/150 (99.3%)	ND	99.3	89	Sundar et al., 2006
		Blood, n = 49	46/49 (93.8%)	ND	95.3	62.7	Diro et al., 2007
		Blood, n = 100	99/100 (99%)	ND	99	95	Boelaert et al., 2008
		Blood, n = 94	94/94 (100%)	ND	100	87	Mandal et al., 2008
		Blood, n = 100 (Pre); n = 50 (post)	98/100 (98%)	25/50 (50%)	98	100	Singh et al., 2009
		Blood, n = 624	251/624 (40.2%)	ND	91	93	Matlashewski et al., 2013
		Blood, n = 145	105/145 (72.4%)	ND	72.4	99.6	Moura et al., 2013
		Blood, n = 231	204/231 (88.3%)	ND	95.8	98.7	Gao et al., 2015
		Blood, n = 38	25/38 (65.8%)	VL Relapsed, 10/10	67	85	Mondal et al., 2019
		VL Relapsed, n = 10		(100%); T/t Failure, 4/7			
		T/t Failure, n = 7		(57.1%)			
		Blood, n = 51	49/51 (96.1%)	ND	83	ND	Freire et al., 2019
		Blood, n = 128	115/128 (89.8%)	ND	83–94	90.5–98.8	Sanchez et al., 2020
		-do-	116/128 (90.6%)	ND	84.3–94.6	96.5–100.0	-do-
		Saliva, n = 128	94/128 (73.4%)	ND	65–80.3	89.5–98.0	-do-
		Blood, n = 98	73/98 (74.5%)	ND	80	82	Chappuis et al., 2003
		Blood, n = 200	189/200 (94.5%)	ND	88.0–95.4	95.7–100	Mukhtar et al., 2015
	rk28	Urine, n = 87	82/87 (94.3%)	ND	94.3	87.1–97.2	Ghosh et al., 2016
		Blood, n = 87	85/87 (97.7)	ND	97.7	91.9–97.2	-do-
		Blood, n = 219	186/219 (84.9%)	ND	77	96	da Silva et al., 2018
	rKE16	Blood, n = 131	104/131 (79%)	ND	ND	ND	Mbui et al., 2013
		Blood, n = 200	191/200 (95.5%)	ND	92–98	91–100	Vaish et al., 2012
	rK39 (IgG/IgM)	Blood, n = 186	107/186 (57.5%)	ND	91.2	95.3	Freire et al., 2018
DAT	Parasite promastigote	Blood, n = 184	cut-off titre (1:400) 139/184 (75.5%)	ND	97	ND	Chappuis et al., 2003
		Blood, n = 150	≥1:1,600. 147/150 (98%)	ND	96	96.5	Singh et al., 2005
		Blood, n = 15	≥1:1,600; 12/15 (80%)	ND	94–97	85	Adams et al., 2012
		-do-	≥1:3,200: 11/15 (73.3%)	ND	99	100	-do-
		Blood, n = 110	≥1:800; 109/110 (99.1%)	ND	60	ND	Oliveira et al., 2013
		Blood, n = 405	No cut off stated: 246/405 (60.7%)	ND	93–95.2	97.5	Bangert et al., 2018
		Blood, n = 49	≥1:3,200; 46/49 (93.8%)	ND	97	62–65.3	Diro et al., 2007
		Blood, n = 285	252/285 (88.4%)	ND	84.1–92.0	89.2–98.1	Machado de Assis et al., 2012
Dipstick test	LAg	Blood, n = 463	449/463 (96.9%)	ND	ND	ND	Ejazi et al., 2019 Ejazi et al., 2016
	LAg	Urine, n = 97	93/97 (95.8%)	ND	95.8	100	
	rGP63		ND				do
	rCPA		ND				do
	SLA		ND				do
Lateral Flow Device	β-tubulin and LiHyp1	Blood, n = 24	20/24 (83.3%)	ND	90	96–100	Humbert et al., 2019
IFAT	IgG	Blood, n = 405	114/405 (28.1%)	ND	79.4	99	Bangert et al., 2018
		Blood, n = 104	103/104 (99%)	ND	100	99	Cañavate et al., 2011
		Blood, n = 285	251/285 (88.5%)	ND	84.0–92.0	75.0–88.2	Machado de Assis et al., 2012

**Table 2:** Nucleic Acid Markers of VL.

Methods	Target	Biological material	Parasite load Pre T/t (% +ve)	Parasite load Post t/t	Sensitivity(%)	Specificity(%)	References
PCR followed by Southern Blotting	*Leishmania* kDNA	Blood (n = 51)	n = 49/51 (96%)	ND	96	96	Salotra et al., 2001
qPCR	*Leishmania* kDNA	Blood, n = 51	*837 parasites/ml	*LAmB, 1 parasite/ml (day 60)	96.5	100	Mary et al., 2004
		Blood, n = 147	*70,560 parasites/ml	*LAmB, 1 parasite/ml (day 5)	95	100	Mary et al., 2006;
		Blood, n = 31	*8,372 parasites/ml	*AmphoB, 1 parasite/ml (day 30)	ND	ND	Verma et al., 2010
				*SAG: 290 parasites/ml (day 28) *LAmB, 72 parasite genomes/ml (day 7)			
		Blood, n = 46	*894 parasite genomes/ml	*8 parasite genomes/ml (day 30)	100	ND	Sudarshan et al., 2011
		Blood, n = 40	*574.5 parasites genomes/ml	ND	100	ND	Sudarshan et al., 2015
		Blood, n = 40	*192.69 parasites/ml	*LAmB, 0.1 parasites/ml (days not stated)	100	ND	Hossain et al, 2017
		Blood, n = 59	n = 13/59 (22%)	ND	ND	ND	Abbasi et al., 2013
			1–10 parasites/ml				
			n = 23/59 (39%)				
			10–100 parasites/ml				
			n = 19/59 (32%)				
			1,000 and above parasites/ml				
PCR	18s rRNA	Blood, n = 140	129/140 (92.1%)	ND	92.1	99.64	Deborggraeve et al., 2008
		Bone Marrow, n = 170	158/170 (92.9%)	ND	92.9	94.60	
		Blood, n = 500	439/500 (87.8%)	ND	87.8	99.64	Srivastava et al., 2011
		Buccal swab, n = 148	123/148 (83.1%)	ND	83	90.56	Mehrotra et al., 2011
	OligoC- Test	Blood, n = 84	81/84 (96.4%)	ND	96.4	88.8	Basiye et al., 2010
		Blood, n = 79	76/79 (96.2%)	ND	96.2	90	Saad et al., 2010
		Splenic Aspirate, n = 31	30/31 (96.8%)	ND	96.8	90	-do-
		Bone Marrow, n = 64	62/64 (96.9%)	ND	96.9	90	-do-
	Nucleic acid sequence-based amplification oligochromatography	Blood, n = 84	67/84 (79.8%)	ND	79.8	100	Basiye et al., 2010
		Blood, n = 79	76/79 (96.2%)	ND	96.2	100	Saad et al., 2010
		Splenic Aspirate, n = 31	30/31 (96.8%)	ND	96.8	100	-do-
	(NASBA-OC)	Bone Marrow, n = 64	61/64 (96.4%)	ND	95.3	100	-do-
Loop Mediated Isothermal	*Leishmania* kDNA and 18s rRNA	Splenic aspirate, n = 75	68/75 (90.7%)	ND	90.7	100	Khan et al., 2012
Amplification LAMP		Splenic aspirate, n = 15	53/55 (96.3%)	ND	100	98.5	Verma et al., 2013a
		Buffy Coat, n = 84	80/84 (95.2%)	ND	97.65	99.01	Mukhtar et al., 2018
		Buffy Coat, n = 50	46/50 (92%	ND	92	100	Adams et al., 2018
		Blood, n = 55	53/55 (96.4%)	ND	96.4	98.5	Verma et al., 2013a
		Blood, n = 47	44/47 (93.6%)	ND	93.6	100	Ghasemian et al., 2014
		Blood, n = 38	35/38 (92.1%)	ND	97	100	Abbasi et al., 2016
		Blood, n = 66	64/66 (96.9%)	ND	96.9	100	Verma et al., 2017
		Blood, n = 179	176/179 (98.3%)	ND	98.32	96.59	Dixit et al., 2018
Direct Blood Lysis- LAMP	*Leishmania* kDNA and 18s rRNA	Blood, n = 72	67/72 (93%)	ND	93.06%	100	Dixit et al., 2018
Recombinase Polymerase Amplification Assay	*Leishmania* kDNA	Blood, n = 23	23/23 (100%)	ND	100	ND	Mondal et al., 2016
Gene sequencing	*Leishmania* kDNA	Bone marrow, n = 22 Blood, n = 16	20/22 (90.9%) 11/16 (68.7%)	ND ND	91 69	100 100	Hu et al., 2000 -do-
PCR-High resolution melting HRM	*Leishmania* kDNA	Bone Marrow, n = 30	28/30 (90.9%)	ND	ND	ND	Pita-Pereira et al., 2012

### Monitoring of VL

#### Circulatory Biomarkers

In Leishmaniasis, the cytokine microenviroment stimulating polyclonal B cell activation lead to an enhanced isotype switching to IgG1 and IgG3 (Chatterjee et al., 1998; Anam et al., 1999; Bhattacharyya et al., 2014). In addition, soluble lymphokines secreted by T-cells regulate human B cell proliferation and differentiation, along with a predominant Th2 presence that translates into an enhanced presence of anti-leishmanial antibodies (Mukhopadhyay et al., 2012). This has been exploited to develop serological tests for VL with a view to replace invasive procedures for demonstration of parasites in giemsa stained tissue aspirates from spleen, bone marrow, or lymph nodes (Sundar and Rai, 2002).

ELISA based assays using crude or soluble antigens sourced from promastigotes or axenic amastigotes have been used for serodiagnosis of VL, with the rK39-Immunochromatographic test (ICT) being the most robust (Sundar et al., 2006 and references therein; **Table 1**). Additionally, the heightened anti-leishmanial IgG and its subclasses have helped identify active VL, and furthermore, reduction of IgG1 titres was associated with successful treatment (Saha et al., 2005; Ansari et al., 2008; Bhattacharyya et al., 2014). However, the ICT/ELISA technique failed to detect HIV/VL co-infected cases, possibly owing to their immunosuppressed status (Medeiros et al., 2017). Another limitation was that the ICT showed positivity in a significant proportion of apparently healthy individuals in endemic regions as also remained positive for long periods after cure from VL (Das et al., 2020). Other *Leishmania* specific coating antigens are recombinant GP63 (rGP63), recombinant cysteine protease A (rCPA), and soluble leishmania antigen (SLA, Ejazi et al., 2016).

To circumvent the false positivity in patients cured from VL most likely due to the presence of anti-leishmanial antibodies, detection of antigen in urine is an excellent alternative and can be expected to broadly correlate with the parasite load. Till date, target antigens tested by the ELISA method (**Table 1**) include recombinant GP63 (rGP63), recombinant cysteine protease A (rCPA) and SLA (Ejazi et al., 2016). A major advantage of this approach is its potential to be implemented in a field setting. However, these antigen based tests have shown moderate sensitivity (**Table 1**), and efforts should therefore be focussed on improving their sensitivity, as also evaluate their potential as a “test of cure”.

#### Nucleic Acid Markers in VL

Nucleic acid based methods are gaining popularity, especially for monitoring treatment effectiveness, a point very relevant for ensuring success of the Leishmaniasis elimination program. Till date, several molecular based assays such as PCR or quantitative PCR (qPCR) have been validated and serve as reference tools for diagnosis of Leishmaniasis (Salotra et al., 2003; Sundar et al., 2018). The widespread availability of these tests at peripheral health centres to diagnose VL could have a great impact on disease management.

To enable *Leishmania* detection, a molecular target should have high abundance and this criteria is best achieved by (i) kinetoplast mini-circle DNA (kDNA), present as 1,000s copies per cell in all *Leishmania* sp (Salotra et al., 2001; Mary et al., 2004; Mary et al., 2006; Verma et al., 2010) and (ii) 18s rRNA (Deborggraeve et al., 2008; Mehrotra et al., 2011; Srivastava et al., 2011). As a diagnostic tool for VL, the kDNA based qPCR or real-time PCR (**Table 2**) has stood the test of time (Mary et al., 2006; Verma et al., 2010; Sudarshan et al., 2011; Abbasi et al., 2013; Sudarshan et al., 2015; Hossain et al., 2017). However, its assessment for quantification of relapse or treatment response remain limited (Mary et al., 2006; Verma et al., 2010; Sudarshan et al., 2011; Sudarshan et al., 2015). It is essential that these qPCR approaches be applied to address the last mile challenges of the South Asia Leishmaniasis elimination program (**Table 2**).

Taken together, it is reasonable to propose that PCR methods could perhaps replace the conventional microscopic detection of LD bodies in giemsa stained tissue aspirates for monitoring of VL, the latter approach being fraught with limitations that include invasiveness and low sensitivity, especially post treatment. However, molecular tests carry the burden of cost, time, necessity for specialized personnel and equipment, as also a stable cold chain to minimize chances of denaturation of reagents and samples. It can be envisaged that following the COVID-19 pandemic, upgrading of the government funded molecular diagnostic facilities could potentially be harnessed for control programs pertaining to Neglected Tropical Diseases such as VL and PKDL.

An emerging option translatable to a field scenario is the Loop-mediated isothermal amplification (LAMP) assay (**Table 2** **)**, which includes a quick and easy DNA extraction method, such as “boil and spin” (Notomi et al., 2000). However, the main issue with LAMP is false positivity as also the assay requires the use of six primers which increases the possibility of a primer-dimer formation. Bst DNA polymerase is commonly used in LAMP because they have strong strand displacement activity (required for isothermal techniques), but being unstable >70°C, it cannot be used in conventional PCR where the denaturation step is close to 90°C (Vink et al., 2018; Nzelu et al., 2019). Considering its field applicability, studies pertaining to the efficacy of LAMP assay in monitoring treatment should be undertaken.

Another promising approach is the recombinase polymerase amplification (RPA) assay used for detection of VL, developed in the format of a mobile suitcase laboratory and could be effective in a resource limited setting (**Table 2**). However, the test needs validation on a larger sample pool, as also its ability to monitor treatment outcome needs to be evaluated.

#### Circulatory and Nucleic Acid Biomarkers in HIV-VL Co-Infection

The detection of anti-leishmanial antibodies in HIV-VL co-infected patients is challenging as the associate immunological dysfunction accounts for the lowered sensitivity of serological tests (Cota et al., 2012). In resource limited settings, DAT and immunoblotting as compared to ELISA and the immunofluorescence antibody test have shown moderate sensitivity (81 and 84%, respectively, Salotra et al., 1999; ter Horst et al., 2009, **Table 3** **)**. The low antibody titres could be augmented using recombinant polypeptides, but requires optimization (Lindoso et al., 2018). In studies where confirmation of VL infection was done by microscopy, the antibody tests showed varied sensitivity (Mathur et al., 2006; Redhu et al., 2006; Sinha et al., 2006), emphasizing the need for molecular based assays. Till date, PCR has often been applied as the primary method of detection (Cota et al., 2012), and amplification of 18s rRNA and kDNA regions have proven to be the best option. Real-time PCR has been adopted for diagnosis of HIV-VL co-infected patients (**Table 3** **)**, where a high parasite burden was reported, but the study population was relatively small (Molina et al., 2013). Further studies are required to monitor the treatment efficacy of these HIV-VL cases.

**Table 3:** Antibody and Nucleic Acid Markers of HIV-VL.

Methods	Target	Biological Material	Disease Presentation	Post t/t (period of follow up) and relapses detected	References
DAT	Crude *Leishmania* antigen	Blood, n = 91	Cut off titre: 1:800; 84/91 (92.3%)	ND	Hailu and Berhe, 2002
		Blood, n = 11ss	Cut off titre : ≥1:51,200; 6/11 (54.5%)	ND	Abass et al., 2015
		Blood, n = 76	Cut off titre: ≥1:3,200; 69/76 (90.7%)	ND	Bangert et al., 2018
ELISA	rk39	Blood, n = 11	9/11 (81.8%)	ND	Abass et al., 2015
		Blood, n = 55	28/55 (51%)	ND	Deniau et al., 2003
	rKLO8	Blood, n = 11	4/11 (36.3%)	ND	Abass et al., 2015
		Blood, n = 77	24/77 (31.1%)	ND	Freire et al., 2019
Immunoblotting	leishmanial antigens 14 and 16 kDa	Blood, n = 28	14/28 (50%)	ND	Fisa et al., 2002
	leishmanial antigens 63 to 66 kD	Blood, n = 35	14/35 (40%)	ND	Santos-Gomes et al., 2000
IFAT	Soluble leishmania antigen (SLA)	Blood, n = 55	30/55 (54.5%)	ND	Deniau et al., 2003
		Blood, n = 76	61/76 (80.2%)	ND	Bangert et al., 2018
		Blood, n = 77	36/77 (46.7%)	ND	Freire et al., 2019
		Blood, n = 18	11/18 (61.1%)	ND	Antinori et al., 2007
		Blood, n = 38	19/38 (50%)	ND	Cruz et al., 2002
ICT	rk39	Blood, n = 11	9/11 (81.8%)	ND	Abass et al., 2015
		Blood, n = 31	21/31 (67.7%)	ND	da Silva et al., 2018
		Blood, n = 76	51/76 (67.1%)	ND	Bangert et al., 2018
		Blood, n = 77	38/77 (49.3%)	ND	Freire et al., 2019
		Blood, n = 55	9/55 (16.3%)	ND	Deniau et al., 2003
	rKE16	Blood, n = 11	7/11 (63.6%)	ND	Abass et al., 2015
PCR	18SSU-rRNA	Blood, n = 19	6/19 (31.58%)	T/t not stated (till 3 years): Relapse, n = 6	Lachaud et al., 2000
		Blood, n = 17	17/17 (100%)	LAmB, (till 1 year): Cured, n = 11; Relapse, n = 6	Antinori et al., 2007
		Blood, n = 27	9/27 (33.33%)	AmphoB with HAART (till 4 years): Cured, n = 2; Relapse, n = 2; Death, n = 5	Buorgeois et al., 2008
Ln-PCR	18SSU-rRNA	Splenic Aspirate, n = 30	26/30 (86.67%)	SAG, (till 1.5 years): Cured, n = 5; Relapse, n = 21	Cruz et al., 2002
		Blood, n = 20	15/20 (75%)	SAG or LAmB, (till 2 years): Cured, n = 6; Relapse, n = 9	Riera et al., 2004
qPCR	*Leishmania* kDNA	Bone Marrow, n = 37	50 parasites/ml	LAmB, (till 4 months): Relapse, n = 25, 40 parasites/ml	Molina et al., 2013

### Monitoring of PKDL

#### Clinical Biomarkers

As the confirmatory diagnosis of PKDL requires a skin biopsy, it is often not performed, and decisions are made based on clinical assessment and a past history of VL (Ganguly et al., 2010). Based on the lesion types, cases with hypopigmented macules are considered as macular PKDL, whereas cases with an assortment of papules, nodules, macules, and/or plaques are termed as polymorphic PKDL (Zijlstra et al., 2003). Irrespective of the geographic region, the differential diagnosis of PKDL includes leprosy, vitiligo, pitryasis alba, and miliaria rubra (Zijlstra, 2019). This can lead to misdiagnosis especially in cases where there is no previous history of VL (Das et al., 2011; El Hassan et al., 2013; Ramesh et al., 2015a). In Africa (mainly Sudan), maculopapular rashes are commonest (90% of cases) and in advanced cases, the papules coalesce to form nodules or plaques that can be confused with leprosy, vitiligo, pityriasis versicolor, tinea corporis, tinea barbae, pityriasis alba (Zijlstra, 2019). Presently, a major challenge is the inability to detect LD bodies in macular cases, which in recent studies have been shown to constitute a substantial component of the burden of PKDL (Zijlstra et al., 2017; Sengupta et al., 2019). Furthermore, in macular cases as hypopigmentation persists even after parasite clearance, it endorses the need for developing an objective and quantifiable “test of cure”.

#### Circulatory Biomarkers

Among the serological tests for PKDL, the rK39 ICT is the most sensitive, rapid, field applicable, and cost-effective tool. However, it is not completely reliable, since a positive ICT could be attributed to a past episode of VL. Similarly, DAT has also been found to be applicable in field conditions, but shares the same disadvantage as ICT (**Table 4**). Another challenge is the microscopic detection of parasites in PKDL lesions (Singh et al., 2015) especially the macular variant (Mondal et al., 2010; Nasreen et al., 2012; Verma et al., 2013a; Bhargava et al., 2018; Ghosh et al., 2018).

**Table 4:** Antigen/Antibody Markers of PKDL.

Methods	Target	Biological source	PKDL Variants	Pre t/t	Post t/t	Sensitivity (%)	Specificity (%)	Reference
ICT	rk39	Blood	All types, n = 50	50/50 (100%)	ND	100	100	Verma et al., 2013a
		-do-	Polymorphic, n = 74	70/74 (91%)	ND	91	100	Salotra et al., 2003
			Macular, n = 14	11/14 (78.6%)	ND			
		-do-	All types, n = 32	30/32 (93.7%)	ND	67	85	Mondal et al., 2019
		-do-	All types, n = 6	6/6 (100%)	ND	100	100	Mathur et al., 2006
		-do-	All, n = 35	35/35 (100%)	ND	100	100	Topno et al., 2018
		-do-	Polymorphic, n = 20	19/20 (95%)	ND	96	100	Das et al., 2007
			Macular, n = 5	5/5 (100%)				
DAT	*Leishmania* Promastigote	Blood	Polymorphic, n = 46	≤1:6,400	ND	1:400: 98.5	1:400: 84.2	Singh et al., 2005
		-do-	Macular, n = 22	Poly: 40/46 (86.9%)		1:800: 98.5	1:800: 96	-do-
				Mac: 13/22 (59.1%)		1:1,600: 94.2	1:1,600: 100	-do-
						1:3,200: 77.2	1:3,200: 100	-do-
			Polymorphic, n = 20 Macular, n = 25 papuloerythematous n = 15	1:800–1:12,800 Poly: 19/20 (95%)	ND	ND	ND	Verma et al., 2015
				Mac: 21/25 (84%)				
				Papuloerythematous: 14/15 (93.3%)				
Subclass ELISA (IgG, IgG1, IgG2, and IgG3)	*Leishmania* Promastigote	Blood	Polymorphic, n = 20	IgG: 23/23 (100%), cut off OD = 0.27	ND	IgG = 100	IgG = 96;	Saha et al., 2005
			Macular, n = 3	IgG1: 21/23 (91.3%), cut off OD = 0.48		IgG1 = 91	IgG1 = 96.7;	-do-
				IgG2: 10/23 (43.5%), cut off OD = 0.45		IgG2 = 45.5	IgG2 = 93.3;	-do-
				IgG3: 19/23 (82.6), cut off OD = 0.25		IgG3 = 81.8	IgG3 = 83	-do-
		Blood	Polymorphic, n = 41 n = 22 (end of t/t)	IgG: Poly: 0.62 [0.27–0.92],	Poly : IgG1 and IgG3 levels	ND	ND	Mukhopadhay et al., 2012
				Mac: 0.36 [0.17–0.56],				
				IgG1: Poly: 0.41 [0.26–0.87]	decreased significantly (p < 0.01 and	ND	ND	-do-
				Mac: 0.33 [0.12–0.41]				
			Macular, n = 16 pr n = 6post (end of t/t)	IgG2: Poly: 00.08 [0.00–0.16]	p < 0.001	ND	ND	-do-
				Mac: 0.11 [0.04–0.22]				
				IgG3: Poly: 0.55 [0.20–0.61]	Mac: neither IgG1 nor IgG3 levels decreased	ND	ND	-do-
				Mac: ND				
Glyco CIC ELISA	*Leishmania* promastigote	Blood	Polymorphic (32)	81/86 (94.2%): 0.46 ± 0.03	ND	95.6	99.3	Jaiswal et al., 2018
	(9-O-acetylated sialic acids containing Immune complex)		Macular (54)	0.069 ± 0.002 (EC, n = 19)				
				0.062 ± 0.001 (NEC, n = 34)				
Gal 1 ELISA	Poricin	Blood	Polymorphic (n = 32)	Polymorphic:	ND	GAL1 ELISA:	GAL1 ELISA	Datta et al., 2019
			Macular (n = 37)	IgG GAL1 27/32 (84.4%)		98.5	: 91	
				IgG GAL2 31/32 (96.9%)				
				IgG subclasses ND				
Gal2	Asialofetuin	-do-	-do-	Macular:				
ELISA				IgG GAL1 27/39 (69.2%)	ND	GAL2 ELISA:	GAL2 ELISA	-do-
				IgG GAL2 ELISA, 37/39 (94.9%)		95.7	: 98.1	
				IgG subclasses ND				
ADA level by ELISA	Adenosine Deaminase (ADA)	Blood	n = 34 (Pre and Post)	Significantly higher than healthy controls (p < 0.05)	Significantly reduced by 2.3-fold	ND	ND	Vijayamahantesh et al., 2016

#### Nucleic Acid Biomarkers

**Figure 2:**
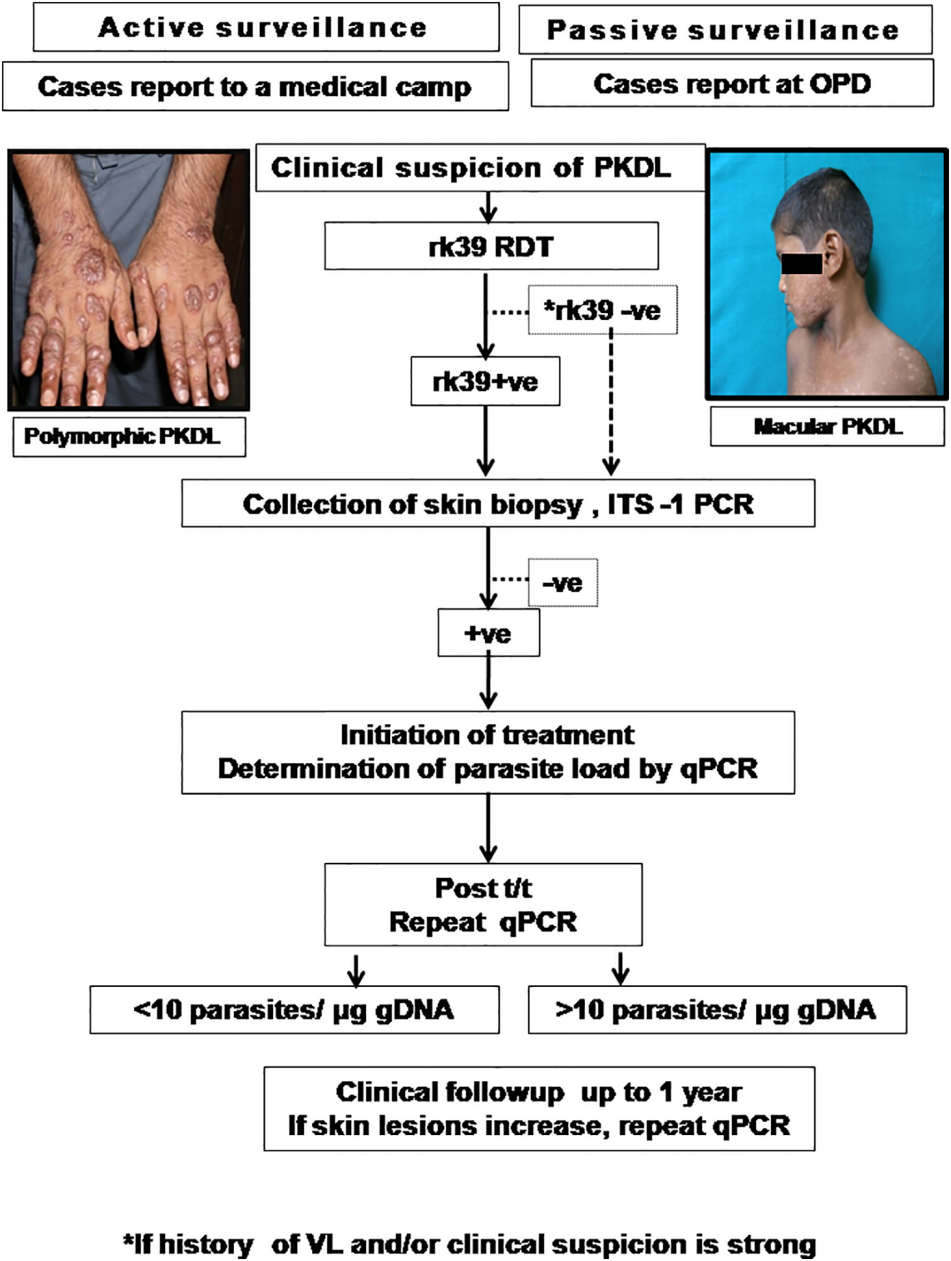
Proposed algorithm for diagnosis of PKDL.

A confirmed diagnosis of PKDL is necessary for clinical trials, and is currently based on the detection of *Leishmania* parasites by microscopy in a slit skin smear. Generally, parasites are present in most papulonodular lesions but in macular lesions, the positivity rate can be as low as 20–40% (Ramesh et al., 2015a; Verma et al., 2015). Several target sequences have been used for the PCR like ribosomal RNA genes, kinetoplast DNA (kDNA), mini-exonderived RNA (med RNA) genes and genomic repeats, the β-tubulin gene region, glycoprotein 63 (gp63) gene locus, internal transcribed spacer (ITS) regions and has been endorsed as a more sensitive method (Salotra et al., 2003 and references therein). Previously, a standardized real time PCR assay, based on Taqman chemistry that targeted the conserved REPL-repeat region of the *Leishmania* genome was used to assess the infection/disease dynamic in asymptomatic *L*. *donovani*-infected individuals (Mondal et al., 2016, **Table 5**). Although kDNA based qPCR or real-time PCR has allowed for detection and quantification of the number of parasites at disease presentation (**Table 5**), it is equally important that longitudinal studies be performed to evaluate the qPCR as a tool to quantify the parasite clearance post-treatment (Ramesh et al., 2011; Verma et al., 2013a; Ramesh et al., 2015b; Bhargava et al., 2018; Moulik et al., 2018). Accordingly, we propose an algorithm for monitoring parasite kinetics (**Figure 2**).

**Table 5:** Nucleic Acid Markers of PKDL.

Methods	Target	Tissue source	PKDL variant	+ve at disease presentation,parasite load, (% +ve)	Parasite loadPost T/t	Sensitivity(%)	Specificity(%)	Reference
PCR	kDNA	Skin biopsy	n = 48	45/48	ND	93.8	100	Salotra et al., 2001
		Skin biopsy	n = 25	24/25	ND	96.0	100	Salotra et al., 2003
PCR	18s rRNA	Skin biopsy	n = 25	24/25	ND	ND	ND	Srivastava et al., 2011
qPCR	Leishmania *kDNA*	Skin biopsy	Macular, n = 2	Pooled, n = 26	Miltefosine (Day 84)	100	ND	Ramesh et al., 2011
			Indurated, n = 11	667 (3–240,000)/μg gDNA	; Pooled, n = 15			
			Polymorphic, n = 13		All were -ve			
		Slit aspirate	Nodular, n = 26	9,790 parasites/μl slit aspirate	SAG or Miltefosine (Day 30); Pooled, n = 19;	100	100	Verma et al., 2013a
			Papular/Macular, n = 24	427 parasites/μl slit aspirate				
		Skin Biopsy	Nodular, n = 26	38,205 parasites/μg of tissue DNA	17/19 (89.5%) were negative	ND	ND	-do-
			Papular/Macular, n = 20	599 parasites/μg of tissue DNA				
		Slit aspirate	Polymorphic, n = 59 Macular, n = 14 1 month post t/t	62/73 (84.9%): 2,302 parasites/μl slit aspirate 11/73 (15%): 11,842 parasites/μl slit aspirate -ve, n = 26/30 (86.7%) Residual parasites in 2/30 (6.7%), cured n = 2/30 (6.7%), relapsed	<10 parasites/μl slit aspirate <10 parasites/μl slit aspirate	ND	ND	Ramesh et al., 2015b
			12 months post t/t	Relapsed: n = 3/73 (4.1%)				
			18 months post t/t	Relapsed: n =11/73 (15%)				
		Slit Aspirate	Macular, n = 4	60 parasites/μl	ND	77	ND	Bhargava et al., 2018
			Papular, n = 20					
			Nodular, n = 26					
		Skin Biopsy	Macular, n = 4	502 parasites/μg gDNA	ND	ND	ND	-do-
			Papular, n = 17					
			Nodular, n = 26					
		Skin biopsy	Macular, n = 91	3,665 (615–21,528)/μg gDNA	Miltefosine (day 84): Macular, n = 17, <10/μg gDNA Polymorphic, n = 21, <10/μg gDNA LAmB (day 21) 2,128 (544–5,763)/μg gDNA 2,541 (650–9,073)/μg gDNA	ND	ND	Moulik et al., 2018
			Polymorphic, n = 93	18,620 (1,266–93,934)/μg gDNA				
	*Leishmania* REPL repeats (L42486.1)	Skin biopsy	All types, n = 20	20/20 (100%)	ND	100	100	Mondal et al., 2016
		Skin biopsy	Macular, n = 38	34/40 (85%)	Miltefosine (day 84) qPCR: n = 3 +ve	85	100	Hossain et al., 2017
			Papular, n = 2	295.46 (1.38–4,065.89) parasites/μg gDNA				
		Skin biopsy	Macular, n = 91	83/91 (91.21%)	ND	50.6	100	Ghosh et al., 2018
		Blood	Macular, n = 91	Negative (n = 91)	ND	91.2	100	
Ln-PCR	SSU-rRNARegion	Blood, buffy coat	Macular, n = 28	14/28 (50%)	ND	ND	ND	Mondal et al., 2010
	*Leishmania* kDNA	Skin biopsy	Popymorphic, n = 21	27/29	ND	93	ND	Sreenivas et al., 2004
			Macular, n = 8					
		Skin biopsy	Macular, n = 74	69/74 (93.2%)	ND	94.5	ND	Nasreen et al., 2012
			Papular, n = 33	32/33 (97%)	ND			
			Nodular, n = 3	3/3 (100%)	ND			
		Skin biopsy	Macular, n = 38	21/40 (52.5%)	Miltefosine (day 84) Ln-PCR: n = 1 +ve	52.5	100	Hossain et al., 2017
			Papular, n = 2					
*L donovani* Recombinase Polymerase AmplificationRPA Assay	*Leishmania* kDNA	Skin biopsy	All types, n = 20	20/20 (100%)	ND	100	100	Mondal et al., 2016
Loop Mediated Isothermal Amplification (LAMP)	*Leishmania* kDNA	Skin Biopsy	All types, n = 62	60/62 (96.8%)	ND	96.8%	ND	Verma et al., 2013b
		Skin Biopsy	All types, n = 67	65/67 (97%)	n = 21, post t/t	97%	ND	Verma et al., 2017
					19/21, -ve			
					2/21 positive			

Patients are enrolled if they present with clinical features suggestive of PKDL either by active case detection and reporting at a medical camp, or presenting in the Dermatology outpatient departments (OPD) of Govt. Medical Colleges. The suspected cases should be examined, and the rK39 strip test performed and if positive, a confirmation by ITS-1 PCR should be performed using a 4mm skin biopsy (Das et al., 2011). In cases where rk39 is negative, but the patient provides a history of VL and/or clinical features are strongly suggestive of PKDL, ITS-1 PCR should be performed using DNA isolated from a skin biopsy. In cases that are ITS1-PCR positive, a kDNA based qPCR should be performed for determination of parasite load at disease presentation (Moulik et al., 2018). After completion of treatment as per guidelines of the National Vector Borne Disease Control Programme (NVBDCP), the parasite load should again be quantified by kDNA based qPCR (Moulik et al., 2018). Patients with a parasite load <10 parasites/µg genomic DNA may be considered as cured, and clinically followed up for possible reappearance of lesions. However, in patients with >10 parasites/µg genomic DNA, they should be monitored closely up to 1 year and if the skin lesions increase, a repeat qPCR done. The parasite load that constitutes complete parasitological clearance remains a pertinent, yet unanswered question and it may be anticipated that implementation of such an algorithm may provide this information (**Figure 2**).

The nested PCR method targeted at minicircle kDNA of *Leishmania* proved to be highly effective and useful for detecting *L. donovani* genes in skin biopsy specimens from patients with PKDL (Nasreen et al., 2012). In PKDL, Mondal et al. (2016) demonstrated the diagnostic potential of the recombinase polymerase amplification (RPA) assay. This test was performed using a mobile suitcase laboratory approach, which endorsed its applicability in field settings (**Table 5**). However, its potential for monitoring the treatment outcome is warranted. Another field-friendly adaptation for DNA-based detection for PKDL cases is the (closed tube) loop-mediated isothermal application (LAMP, Verma et al., 2013b; Verma et al., 2017) but like the RPA assay, its efficacy for monitoring treatment remains to be assessed.

The availability of biomarkers and “test of cure” tools for Leishmaniasis will facilitate not only monitoring of active VL cases but also provide important epidemiological data. Therefore, it is necessary to establish an algorithm that can potentially differentiate between patients progressing to active VL *vis-a-vis* those that remain asymptomatic. Similarly, identification of biomarkers that can predict which cases of VL are likely to develop PKDL would be a value addition for the elimination program.

### Xenodiagnosis in VL and PKDL

Xenodiagnosis is the classical approach for quantifying transmissibility from a host to an insect species with a view to distinguish infectious from non-infectious hosts (Singh et al., 2020). This method when applied can help define the characteristics of *L. donovani* transmission and provide key epidemiological evidence to guide the program. During xenodiagnosis of human VL caused by *L. infantum*, successful transmission to sand flies was confirmed in 6/6 (100%) VL–HIV co-infected patients (Molina et al., 1999). Similarly, in a study in Brazil, 11/44 (25%) VL patients were established as transmitters and importantly, no infections were detected in sand flies allowed to feed on 147 “asymptomatic” subjects (Costa et al., 2000). In an Indian study by Mukhopadhyay and Mishra (1991), one sandfly out of 183 (0.5%) that fed on VL patients during the day time was infected, whereas in sandflies fed similarly at midnight, 4/75 (5%) were infected, suggesting a periodicity for blood or tissue parasitemia.

In order to find a correlation, if any, between sandfly infection rates and parasite load, qPCR was performed using skin and blood sourced from patients with PKDL (Molina et al., 2017). It was further expanded to larger cohorts in Bangladesh wherein the infectiousness was higher in the polymorphic variant (17/21 81%) vs. macular (9/35, 35%) cases (Mondal et al., 2019); similarly, a substantial proportion of VL cases (10/15, 67%) were also able to transmit to sandflies (Mondal et al., 2019). Taken together, as tracking of the *Leishmania* parasite in sandflies provides key information regarding the infectiousness of VL and PKDL cases (Singh et al., 2020), application of molecular tools could provide information regarding the reservoir competence.

## Discussion

The availability and accessibility of new biomarkers and diagnostic tests for Leishmaniasis can facilitate not only the confirmation of active VL and PKDL cases, but also allow for epidemiological studies to be undertaken, identification of asymptomatic individuals, assess the degree of infectiousness to sand flies, and last but certainly not the least, monitor treatment efficacy in an objective and precise manner. It is necessary to establish an algorithm that differentiates between patients progressing to active VL *vis-a-vis* those that remain asymptomatic. Another aspect is the availability of markers for recognising patients with VL who develop PKDL, as this may have a considerable impact on disease control. The biggest hurdle so far is the monitoring of anti-leishmanial therapy and longitudinal studies are necessary to understand the parasite dynamics.

## Concluding Remarks

In Leishmaniasis (particularly VL and PKDL), the biomarkers detailed in this review have been primarily used as diagnostic tools, and many of them have proven to be fairly robust and reproducible. In order to sustain the gains achieved *via* the kala-azar elimination program, the last mile strategies should include (1) validation of tools for monitoring anti-leishmanial therapy, (2) promoting epidemiological surveillance in the post-elimination phase for detection of potential outbreaks, along with (3) supporting research focussed on identification of proxy markers for detecting sandfly infectivity (National Vector Borne Disease Control Programme, 2020).

## Data Availability Statement

The original contributions presented in the study are included in the article/supplementary material. Further inquiries can be directed to the corresponding author.

## Author Contributions

SM, SS, and MC performed the literature search, prepared, and edited the manuscript. All authors contributed to the article and approved the submitted version.

## Funding

This study received financial support from Indian Council for Medical Research (Grant number: 6/9-7[151]2017-ECD II); Dept. of Health Research, Govt. of India (Grant number: DHR/HRD/Fellowship/SUG-05/2015-16); Fund for Improvement of S&T infrastructure in Universities and Higher Educational Institutions (FIST) Program, Dept. of Science and Technology, Govt. of India (DST-FIST) (Grant number: SR/FST/LS1-663/2016); Dept. of Science and Technology, Govt. of West Bengal (Grant number: 969 [Sanc.]/ST/P/S&T/9G-22/2016). MC is a recipient of a JC Bose Fellowship (JCB/2019/000043), Science Engineering & Research Board, DST, Govt. of India and SS a recipient of Junior Research Fellowship, CSIR, Govt. of India.

## Conflict of Interest

The authors declare that the research was conducted in the absence of any commercial or financial relationships that could be construed as a potential conflict of interest.
